# RF-Enabled Deep-Learning-Assisted Drone Detection and Identification: An End-to-End Approach

**DOI:** 10.3390/s23094202

**Published:** 2023-04-22

**Authors:** Syed Samiul Alam, Arbil Chakma, Md Habibur Rahman, Raihan Bin Mofidul, Md Morshed Alam, Ida Bagus Krishna Yoga Utama, Yeong Min Jang

**Affiliations:** Department of Electronic Engineering, Kookmin University, Seoul 02707, Republic of Korea; syed.samiul.alam@ieee.org (S.S.A.); arbilchakma@ieee.org (A.C.); rahman.habibur@ieee.org (M.H.R.); raihanbinmofidul@ieee.org (R.B.M.); mmorshed@ieee.org (M.M.A.); idabaguskrishnayogautama@gmail.com (I.B.K.Y.U.)

**Keywords:** UAV detection, classification, deep learning, convolutional neural network, multiscale architecture

## Abstract

The security and privacy risks posed by unmanned aerial vehicles (UAVs) have become a significant cause of concern in today’s society. Due to technological advancement, these devices are becoming progressively inexpensive, which makes them convenient for many different applications. The massive number of UAVs is making it difficult to manage and monitor them in restricted areas. In addition, other signals using the same frequency range make it more challenging to identify UAV signals. In these circumstances, an intelligent system to detect and identify UAVs is a necessity. Most of the previous studies on UAV identification relied on various feature-extraction techniques, which are computationally expensive. Therefore, this article proposes an end-to-end deep-learning-based model to detect and identify UAVs based on their radio frequency (RF) signature. Unlike existing studies, multiscale feature-extraction techniques without manual intervention are utilized to extract enriched features that assist the model in achieving good generalization capability of the signal and making decisions with lower computational time. Additionally, residual blocks are utilized to learn complex representations, as well as to overcome vanishing gradient problems during training. The detection and identification tasks are performed in the presence of Bluetooth and WIFI signals, which are two signals from the same frequency band. For the identification task, the model is evaluated for specific devices, as well as for the signature of the particular manufacturers. The performance of the model is evaluated across various different signal-to-noise ratios (SNR). Furthermore, the findings are compared to the results of previous work. The proposed model yields an overall accuracy, precision, sensitivity, and $F_{1}$-score of 97.53%, 98.06%, 98.00%, and 98.00%, respectively, for RF signal detection from 0 dB to 30 dB SNR in the CardRF dataset. The proposed model demonstrates an inference time of 0.37 ms (milliseconds) for RF signal detection, which is a substantial improvement over existing work. Therefore, the proposed end-to-end deep-learning-based method outperforms the existing work in terms of performance and time complexity. Based on the outcomes illustrated in the paper, the proposed model can be used in surveillance systems for real-time UAV detection and identification.

## 1. Introduction

In recent times, unmanned aerial vehicles (UAVs), widely recognized as drones, have become an area of substantial interest. Without a pilot on board, UAVs can be operated from miles away with the help of a remote controller. Initially, their applications were limited to military sectors [1]. Military UAVs are used in warfare, surveillance, air strikes, investigations, etc. [2]. However, drones are now being utilized for a diverse range of applications that extend beyond the military, making them a valuable tool in many different industries. For example, governments use UAVs for forestry surveillance [2], disaster management [3], remote sensing [4], etc. Companies such as Amazon, UPS Inc., and many others are using them for their product delivery services [5], etc. In agriculture, drones are being used for spraying fertilizers and insecticides and crop monitoring [4]. Firefighters, healthcare services, and hobbyists are utilizing drones for rescue missions, ambulance services, and recreational photography [2]. UAVs are now widely employed beyond military applications; rather, they are an inherent part of our society. Most UAVs registered in the United States serve recreational purposes, over 70%, while the rest are used for commercial applications [6].

The increased number of drone users raises concerns for privacy and security [7]. The deployment of civilian drones in national airspace has raised concerns about unauthorized and unskilled pilots intruding into restricted zones and disrupting flight systems. Limited regulations during drone purchases can contribute to this issue. For example, a few years ago, a civilian drone crashed into an army chopper [8]. The most concerning issue is about exploiting UAVs for terrorist attacks and illegal surveillance [6]. To prevent the mentioned occurrences, an anti-UAV system capable of detecting, identifying, and neutralizing unauthorized UAVs capturing information utilizing different sensors is desired [9]. Besides, UAV and UAV flight controllers, Bluetooth, and WIFI also use the 2.4 gigahertz (GHz) band. Detecting UAVs among these signals is a very challenging task as those types of signals have become more common in any infrastructure in the present day. Identification and classification involve identifying the model of the received radio frequency (RF) signal. The neutralization involves raising alarms or bringing down the unauthorized UAV or tracking the source of the UAV controller signal. Several works have explored methods of detecting drones using various technologies, including radar, audio, video, thermal imaging, and RF. Radar-based techniques rely on the principle of using electromagnetic backscattering to detect and identify aerial objects by analyzing their radar cross-section (RCS) signature [10]. Due to their smaller size, detecting drones using RCS analysis can be more challenging when compared to airships. In audio-based techniques, a microphone is used to collect the audio fingerprint of the engine and propellers [6,10]. The video surveillance camera is used to monitor areas with the help of computer vision from the visual feature objects (e.g., UAVs). In the thermal-imaging-based system, the thermal signature of the UAV emitted from the engine is used for detection. In RF-based systems, RF signals are intercepted and analyzed for identification and detection. The advantage of the RF-based detection technique is that it can work regardless of any weather condition, as well as day or night. Therefore, RF-based surveillance system has become more promising than other existing systems in recent times. However, one of the major challenges of RF-based sensing is the presence of other 2.4 GHz signals like WIFI and Bluetooth.

Machine learning (ML) and deep learning (DL) techniques have revolutionized many areas such as image segmentation [11,12] and disease detection [13]. With the development of DL algorithms, deep-learning-assisted drone-detection techniques have become popular in the literature. A deep neural network (DNN) was integrated to classify multirotor UAVs with audio signals in [14]. The authors have evaluated different architectures such as recurrent neural network (RNN), convolutional neural network (CNN), and convolutional recurrent neural network (CRNN) and compared the performances of these models against late fusion methods, which performed better than existing solo network architectures. A weight-optimized long short-term memory (LSTM) model was proposed to classify drones using radar cross-section (RCS) signatures at millimeter-wave (mm wave) in [15]. Due to the optimization, the computational overhead was reduced by denying the flow of the gradient through the hidden states of the LSTM layers. Furthermore, adaptive learning rate optimization was also introduced. Previously, signatures of RCS were converted into images that required more computation. The LSTM-ALRO model introduced in this work yielded better results than existing image-based deep learning models. However, the impediments of the audio and radar-based techniques are that they are highly sensitive to noise and their performance suffers with the increase in range. Moreover, radar-based techniques are not effective with smaller drones [10]. The RF-based technique using deep learning for classifying multiple drones was presented in [16]. The authors proposed a supervised deep learning algorithm to perform the detection and classification tasks. They have used short-term Fourier transform (STFT) for preprocessing RF signals. STFT was first used in this work to perform preprocessing of the data, which was fundamental to the increased performance of their algorithm. In [10], the authors presented RF-UAVNet, a convolutional network for the drone surveillance system, to identify and classify drones based on RF signals. The proposed architecture consists of grouped convolutional layers reducing network size and computational cost. DroneRF [17], a publicly available dataset for RF-based drone detection systems, was used in this work. The DroneRF dataset was also used in [18], where authors introduced compressed sensing technology, replacing the traditional sampling theorem, and a multi-channel random demodulator to sample the signal. To detect the UAV, multistep deep learning was used. The DNN was used to detect the UAV and a CNN was used to further identify the UAV. However, while using the DroneRF dataset, considering other signals present at the 2.4 GHz band was not possible [19]. So, Bluetooth and WIFI signals were not considered in [10,16,18]. In [6], the authors performed an analysis of RF-based UAV detection and identification, considering the intrusion of other wireless signals such as Bluetooth and WIFI. They performed continuous wavelet transform (CWT) and wavelet scattering transform (WST) for extracting features. They considered transient and steady states while classifying and identifying the signal. Furthermore, they performed multiple image-based feature extraction techniques to compare the performance with coefficient-based techniques (CWT, WST). They performed several ML models such as support vector machine (SVM), k-nearest neighbors (KNN), and ensemble in combination with principal component analysis (PCA) for classification and identification tasks across various noise levels. They performed transfer learning using SqueezeNet [20], which is a publicly available pretrained model for the classification and identification of UAVs. In this work, the authors only considered drone control signals for detection. However, focusing solely on control signals has a notable limitation when it comes to detecting drones, as these UAVs can be operated from a remote location, potentially rendering them undetectable. Therefore, to get a more reliable outcome, signals transmitted from drones must be considered [19]. Moreover, the authors observed severe performance degradation with lower signal-to-noise ratios (SNR). In [19], the authors proposed a framework for classifying and identifying and for activity recognition. The authors considered commonplace 2.4 GHz signals such as WIFI and Bluetooth, UAV controller signals, and UAV signals. A stacked noise denoising autoencoder (SDAE) was used for denoising to reduce noise and channel effects. After identifying the unmanned aerial system (UAS), UAV controller signal, or UAV, the classification was further performed to know the exact model of the device after extracting the unique features using wavelet packet transform (WPT) and Hilbert–Huang transform (HHT). Only the steady-state signals were considered as the transient signal can be easily affected by channel effects [6]. In [6,19], the Cardinal RF (CardRF) dataset was also used for UAV detection tasks. However, most of the aforementioned literature [6,18,19] heavily relied on separate feature extraction methods and noise reduction methods, which significantly increase the workload and complexity [21].

To mitigate the aforementioned challenges, we propose an end-to-end deep CNN-based model to detect and identify UAS signals in the presence of WIFI and Bluetooth signals with various SNRs. We aim to exploit multiscale convolutional architecture to classify and detect UAV or UAV controller signals. We have used the CardRF [22] dataset for training, as well as for evaluating the predictive performances of the proposed model, as other datasets available for UAV surveillance have some shortcomings, as described in [19]. The stacked convolutional layers in the network-extract-enriched information from the noisy data. Therefore, the proposed model does not require any further denoising or feature-extraction steps. Moreover, the feature-extraction capability of the network is enhanced by the introduction of the multiscale architecture. Features of different scales are obtained by paralleling different convolutional kernels. Residual connections are also inserted in the proposed model to avoid gradient explosion, which results in superior training outcomes. Furthermore, the residual structures and maxpooling improve the performance of the model in backpropagation [23].

In summary, the main contributions of this work are presented as follows:

- An end-to-end DL-based system has been proposed to detect and identify UAS, Bluetooth, and WIFI signals across various different noise levels.
- The model does not require any manual feature extraction steps, which reduces the computational overhead. The model exploits the RF signature of different devices for the detection and identification tasks.
- Stacked convolutional layers along with multiscale architecture have been utilized in the model, which assists in the extraction of crucial features from the noisy data without any assistance from the feature-extraction techniques.
- The performance of the model has been evaluated using different performance matrices (e.g., accuracy, precision, sensitivity, and F1-score) on the CardRF dataset.
- After conducting comparative experiments, we have established that our proposed network outperforms the existing works in terms of performance and time complexity.

The rest of this paper is structured as follows: Section 2 describes the methodology of UAV detection and identification; Section 3 is based on the experimental results, as well as implementational details; and the conclusion was finally drawn in Section 4.

## 2. Methodology

**Figure 1:**
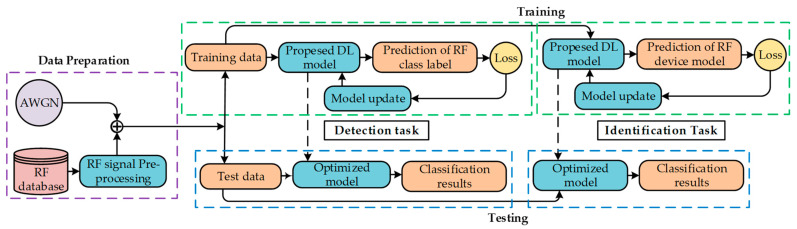
The architecture of the proposed system for UAS signal detection and identification.

This section describes the identification and detection of UAS signals along with Bluetooth and WIFI signals utilizing the proposed architecture using the CardRF dataset. Figure 1 depicts the complete architecture of the proposed system for the UAS signal. The samples sourced from the RF database are preprocessed, and additive white Gaussian noise (AWGN) is incorporated into the samples to generate noisy samples of different SNRs. Each requisite step of UAS signal detection and identification is illustrated in a detailed manner in the following sections.

### 2.1. RF Dataset Description

For the mentioned system, CardRF, a large-scale dataset, is utilized for different RF-based signals (e.g., UAS, WIFI, and Bluetooth) detection and device identification. The dataset contains signals from five UAVs (one Beebeerun (Bbrun), four DJI), five UAV flight controllers (one 3DR and four DJI), five Bluetooth devices (iPad, iPhone, and smartwatch), two WIFI routers (one Cisco and one TP-link). The captured signals were passed through a 2.4 GHz bandpass filter to ensure that they have the same frequency band [19]. Each signal contains five million sampling points at 30 dB SNR. The details of signal acquisition experiments of the signals are given in [19]. In this article, the steady state of the signals with 1024 sampling points per slice is considered. The dataset used in this literature is shown in Table 1 in a detailed manner.

### 2.2. RF Signal Preprocessing

**Figure 2:**
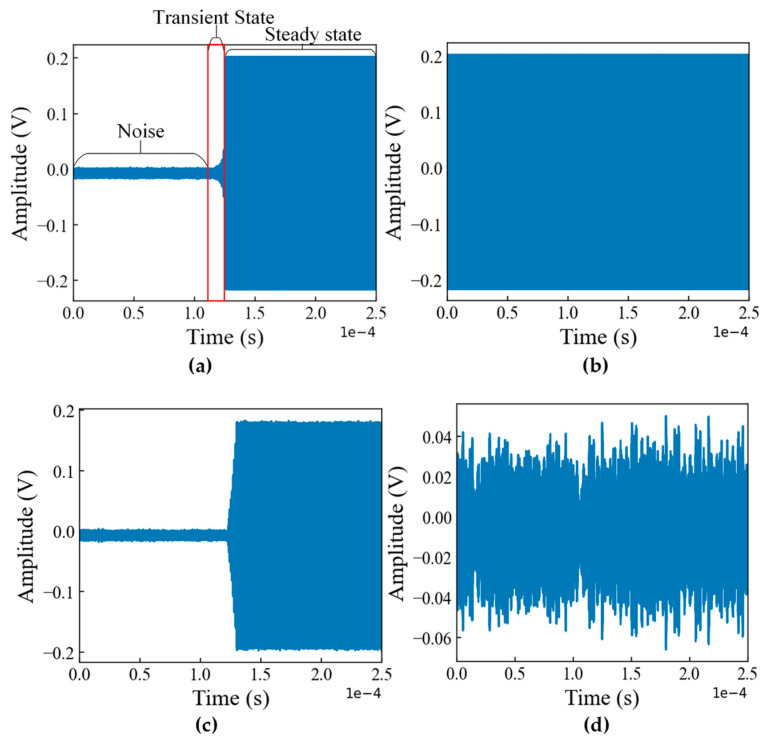
RF signals of (**a**) M600, (**b**) Mavicpro, (**c**) Beebeerun UAV controller, and (**d**) DJI Inspire UAV.

The RF signal pre-processing mentioned in Figure 1 is described here in detail. In the CardRF dataset, each signal contains five million sampling points, which comprise of noise transient state and steady state. In this article, we have considered 10 segments from each signal, where each signal contains 1024 sampling points for the classification tasks, as the minimal length of the signal will introduce enhanced time complexity in the detection and identification system [19]. As some of the classes do contain the transient state, which can be shown in Figure 2, only the steady-state signals were considered. Moreover, the transient state sometimes does not contain reliable features. For this reason, each segment is taken from the steady state and normalized by scaling values in the range of (0, 1) as follows:

(1) $$x_{i_{normalized}}=\frac{x_{i}-x_{i_{min}}}{x_{i_{max}\;}-x_{i_{min}}}$$

where $x_{i\;}$denotes the amplitude of the segmented signal, and $x_{\min}$, $x_{\max}$, and $x_{\mathrm{normalized}}$ denote the minimum, maximum, and normalized amplitude of the signal, respectively.

### 2.3. Noise Incorporation

To investigate the model performance across various noise levels, we have incorporated AWGN to signals to produce noisy signals of 0 dB, 5 dB, 10 dB, 15 dB, 20 dB, and 25 dB *SNR*. To generate noisy signals of desired *SNR*, $SNR_{Target}$, desired noise power, and $P_{Noise_{\;}}$can be calculated using signal power $\;P_{Signal_{\;}}\;$and desired *SNR*, $SNR_{Target}$ as follows:

(2) $${\;P}_{{\mathrm{Signal}}_{\;\mathrm{dB}}}=10\;\log(\frac{\sum_{i=0}^{m}x_{i_{\mathrm{normalized}}\;}}{m})$$

(3) $$\;\;\;\;P_{Noise_{\;dB}}=P_{Signal_{\;dB}}\;SNR_{Target_{\;dB}\;}$$

where *m* denotes signal length, and $\;P_{Signal_{\;dB}}$ is the average signal power in the *dB* unit in Equation (3).$\;P_{Noise_{\;dB}}$ and ${\;\mathrm{SNR}}_{{\mathrm{Target}}_{\;\mathrm{dB}}\;}$are noise power and desired *SNR* in *dB*, respectively. The noise power can be calculated as follows:

(4) $$\;P_{Noise_{\;}}=10^{\frac{\;P_{Noise_{\;dB}}}{10}}$$

where ${\;P}_{{\mathrm{Noise}}_{\;}}$ is the noise power in watts. To produce the noise signal, zero is chosen as the mean noise, $\;P_{Noise}\;$as standard deviation, and the noisy signal is generated using the following equation:

(5) $$X_{i_{Noisy}}=X_{i_{Normalized}}+\eta \left(\mu_{Noise},\;\rho_{Noise}\right)$$

where $X_{i_{Noisy}}$ is the generated noisy signal. η represents the noise signal. $\mu_{\mathrm{Noise}}$ and ${\;\rho }_{\mathrm{Noise}}$ are noise mean and standard deviation, respectively.

**Figure 3:**
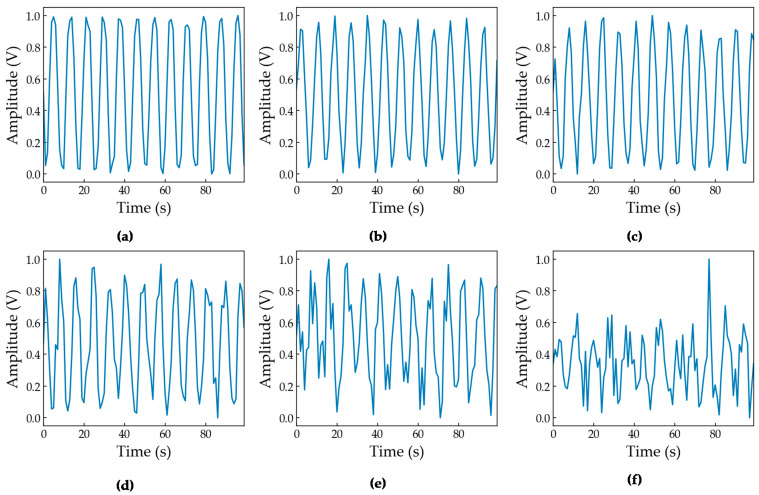
RF signals of a Beebeerun UAV module: (**a**) 25 dB SNR, (**b**) 20 dB SNR, (**c**) 15 dB SNR, (**d**) 10 dB SNR, (**e**) 5 dB SNR, and (**f**) 0 dB SNR.

Figure 3 shows the signal at different noise levels. Figure 3a–c show the signal at 30 dB, 25 dB, and 20 dB, respectively. The difference in RF signal is minimal in these SNRs. However, the quality of the signal degrades with the decrease in SNR, which can be seen in Figure 3e,f.

### 2.4. Model Description

**Figure 4:**
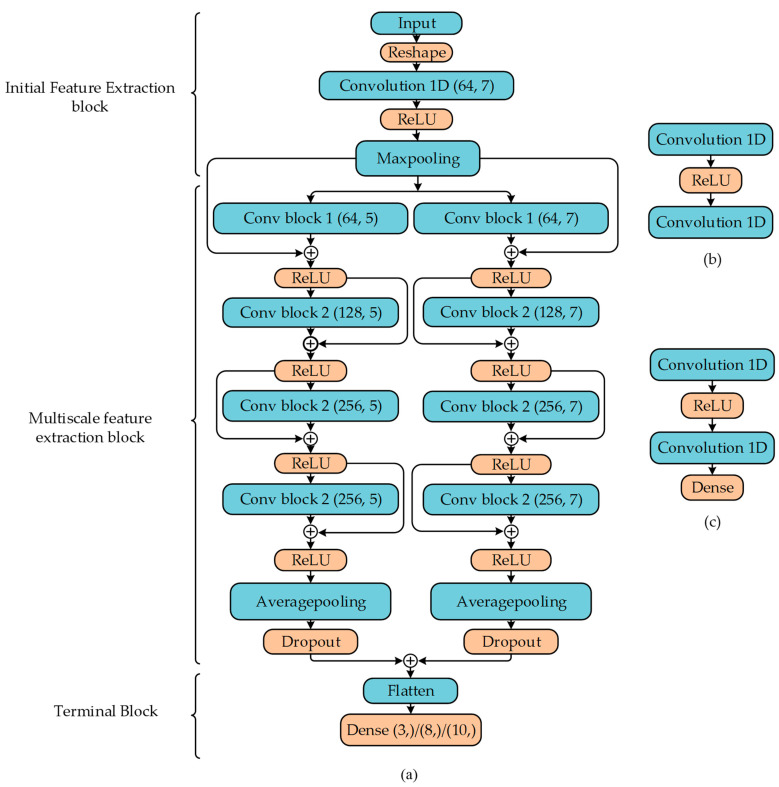
(**a**) Architecture of the proposed multiscale convolutional network model. (**b**) Conv block 1. (**c**) Conv block 2.

Figure 4a describes the complete architecture of the model. The whole model can be divided into three major sections. The first stage is called the initial feature extraction block. At the very top, after the input layer, the one-dimensional data was reshaped to feed into the convolutional layer and followed by a rectified linear unit (*ReLU*) activation function, which is linear for all positive values and zero for all negative values. *ReLU* is computationally inexpensive, which results in less training and inference time. Moreover, it converges faster than other activation functions, such as Tanh. The *ReLU* function can be written as follows:

(6) ReLU(x)=max(x, 0)

Next, the maxpooling layer is used to extract the most prominent features and to reduce the feature map before incorporating multiscale architecture.

The second section is the multiscale feature extraction block. This section consists of both sequential and parallel layers to extract features of the different spatial domains. In our network, we have exploited an architecture with two branches for feature extraction. The architecture of these two branches is identical except for the size of their kernels. Different kernel sizes have been used for experimental purposes. Each branch contains four convolutional blocks (conv block) with different convolutional filters. The first two parallel blocks consist of one convolutional layer followed by a *ReLU* function and another convolutional layer that is described as conv block 1 in Figure 4b. The layers consist of 64 convolutional filters.

(7) $$y_{i}=x_{i}+f\left(x_{i}\right)$$

(8) $$x_{i+1}=ReLU\left(y_{i}\right)$$

where $x_{i}\;$is the output of the maxpooling layer and $f\left(x_{i}\right)$ is the output of the conv block 1. The output of the conv block and maxpooling layers is added, as shown in Equation (8), and passed through the *ReLU* layer, which is the input of the second conv block with 128 filters, which is an instance of conv block 2. The second conv block has the architecture shown in Figure 4c. The difference between this block from the previous one is the output of the second conv layer is passed through a dense layer with *ReLU* activation of 64 units to keep the number of outputs similar to the previous one. The residual block next can be expressed as follows:

(9) $$x_{i+2}=ReLU\left(f\left(x_{i}\right)+f\left(x_{i+1}\right)\right)$$

where $f\left(x_{i}\right)$ and $f\left(x_{i+1}\right)$ are the output of conv blocks. The third and fourth Conv blocks have the same hyperparameters with 256 filters. The outputs of these blocks are passed through a residual block and then the averagepooling layer and dropout layer to reduce overfitting.

The third and final section of the model, which is called the terminal block, contains flatten and *softmax* layers. The outputs of both branches are concatenated and flattened. For the detection task, three classes are used, and for the identification task, ten classes are utilized for specific device identification task and eight for the device manufacturer identification task. However, similar architecture is used for identification and detection tasks except for the *softmax* layer. *Softmax* maps the outputs between zero and one, as well as provides a probabilistic distribution of the likelihood of all the classes. The *softmax* function can be defined as follows:

(10) $$Softmax\left(z_{i}\right)=\frac{e^{z_{i}}}{\sum_{j=1}^{k}e^{z_{j}}}$$

where $z_{i}\;$is the flattened outputs of the previous stage and k is the number of classes. The selection of the number of neurons and layers utilized in this article was based on extensive experimentation. Table 2 depicts a detailed description of the proposed model with the output shapes of each layer, 1, 2, 3, etc., representing the instances of each layer.

## 3. Experimental Results

In this section, implementation details, performance metrics, and model performances are described. Finally, the performance of the proposed model is evaluated with existing work to analyze the effectiveness of the proposed system and unveil its superiority over other existing works.

### 3.1. Implementation Details and Performance Metrics

From the normalized RF signals, 85% of each category is selected for training, and the remaining 15% of the signals are kept for testing purposes for both detection and identification tasks. The total training data number 51,765, and the testing data number 9135 for the detection task, including all three categories. The classifier models are trained using the training data and optimized using an optimizer. Finally, the performance has been evaluated on the testing data (see Figure 1). For the identification task, three classes (iPhone 7, iPad 3, and E5 Cruise) are excluded to compare our work with [6]. The total training data for specific device identification tasks number 43,732, and the testing data number 7718. The training and testing procedures were conducted within an Anaconda Python 3.7 environment on a system featuring a 12th generation Intel Core i7 CPU with a base clock speed of 2.10 GHz, 16 GB of RAM, and a single Nvidia GeForce RTX 3050 GPU with 8 GB of dedicated GPU memory. All the hyperparameters utilized for training the proposed model are shown in Table 3. By varying the noise level, the performance of the proposed model is evaluated, keeping the number of hyperparameters identical. For the cost function, categorical cross-entropy is used for both the detection and identification tasks, which is require multi-class classification. To minimize the loss function, an adaptive moment estimation (Adam) optimizer is used. The benefit of using Adam is that it perceives the learning rate individually for all the parameters. Both the detection and identification models were trained for 120 epochs.

To evaluate the performance of our model, we have computed the accuracy (ACC), precision (PR), sensitivity (SE), and $F_{1}$-score ($F_{1}$), which are also known as evaluation metrics. PR is the ability of the classifier to avoid incorrectly labeling instances as positive if they are truly negative. On the other hand, SE is defined as the ability of the classifier to identify the positive instances. $F_{1}$ is the weighted harmonic mean of both PR and SE. These are defined as follows:

(11) $$ACC_{i}=\frac{TP_{i}+TN_{i}}{TP_{i}+TN_{i}+FP_{i}+FN_{i}}$$

(12) $$PR_{i}=\frac{TP_{i}}{TP_{i}+FP_{i}}$$

(13) $$SE_{i}=\frac{TP_{i}}{TP_{i}+FN_{i}}$$

(14) $$F_{1_{i}}=\frac{2\times PR_{i}\times SE_{i}}{PR_{i}+SE_{i}}$$

where ${\mathrm{TP}}_{i}{,\;\mathrm{TN}}_{i}{,\;\mathrm{FP}}_{i}{,\;\mathrm{and}\;\mathrm{FN}}_{i}$ are true-positive, true-negative, false-positive, and false-negative, respectively, of the ith class. True-positive and true-negative stand for the number of the ith class predicted correctly and the number of other classes that are not predicted as the of ith class, respectively. Whereas false-positive and false-negative are the outcomes that refer to the number of other classes, which are predicted as the ith class and the number of ith classes classified as the other classes, respectively.

### 3.2. Performance Analysis

**Figure 5:**
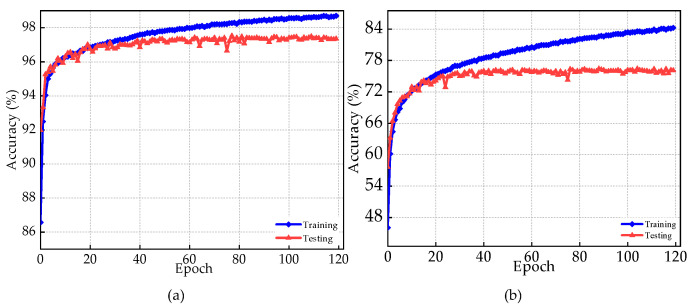
Training and test accuracy curve of the proposed models over 120 epochs for (**a**) RF signal detection task and (**b**) specific device identification task.

In this section, the performance of the proposed model is analyzed across different noise levels for both detection and identification tasks. Figure 5a,b depict the training and testing accuracy curves over 120 epochs for detection and specific device identification tasks, respectively. From the figures, it can be seen that the models do not have overfitting issues. It can also be seen that both models converge rapidly. The training process of the models has been stopped, even though the training accuracy was still improving because of no noticeable improvement in the testing data. The overall training and testing accuracy of the proposed model are 98.7% and 97.53%, respectively, for the detection task, and for the identification task, the model has an accuracy of 76.42%. For the detection task, the accuracy of RF signal detection has higher accuracy as opposed to the specific device identification task, as the model has a higher rate of misclassifying the UAS signals from the devices that are manufactured by the same maker.

We have varied the kernel sizes for the convolutional layers of our models to observe the performance of the model to find the most optimal hyperparameters. Table 4 demonstrates the performance comparison of the proposed model for different kernel sizes. From the table, it can be seen that for the higher SNR values, the accuracy of the model slightly differs, but with the increase in noise, the differences in the performance of the model are more visible. For the detection task, the model shows an accuracy of 98.63% when kernels of size 3 and 7 have been used, which is only 0.01% less than kernel sizes of 5 and 7. However, for 0 dB SNR, the model demonstrates an accuracy of 93.81% with kernel sizes of 5 and 7, which is 0.93% and 0.95% higher than the accuracy of the model with kernel sizes of 3, 7, and 3, 5. The overall accuracy of the model is also higher with 5 and 7 kernel sizes. The same scenario can be seen for the detection task as well. For 0 dB SNR, the accuracy of the model is 88% and 1.81% higher with kernel sizes of 5 and 7 than the models with kernel sizes of 3, 7, and 3, 5. The model yields better results with larger kernel sizes because they reduce false positives and improve accuracy [24]. Moreover, larger kernels also capture more spatial information and extract more relevant features from the noisy signals.

Table 5 shows the overall performance of the model for the detection task in terms of four evaluation metrics numerically using $TP_{i},\;TN_{i},\;FP_{i}$,$\;\mathrm{and}\;FN_{i}$. From the SE metrics, it can be seen that the model can identify 97.53% of UAS signals correctly. The model demonstrates a PR of 98.06%. This high precision rate means the model has a very high rate of $TP_{i}$ in terms of UAS signals. The model also shows a higher SE for UAS signals. These high PR and SE leads to a high $F_{1}$ score as well. The PR, SE, and $F_{1}$ are similar for the UAS and Bluetooth classes. That describes the model can almost accurately classify these two classes. The lower value PR, *SE*, and $F_{1}$ for WIFI can be explained by the fewer training samples of the class.

**Figure 6:**
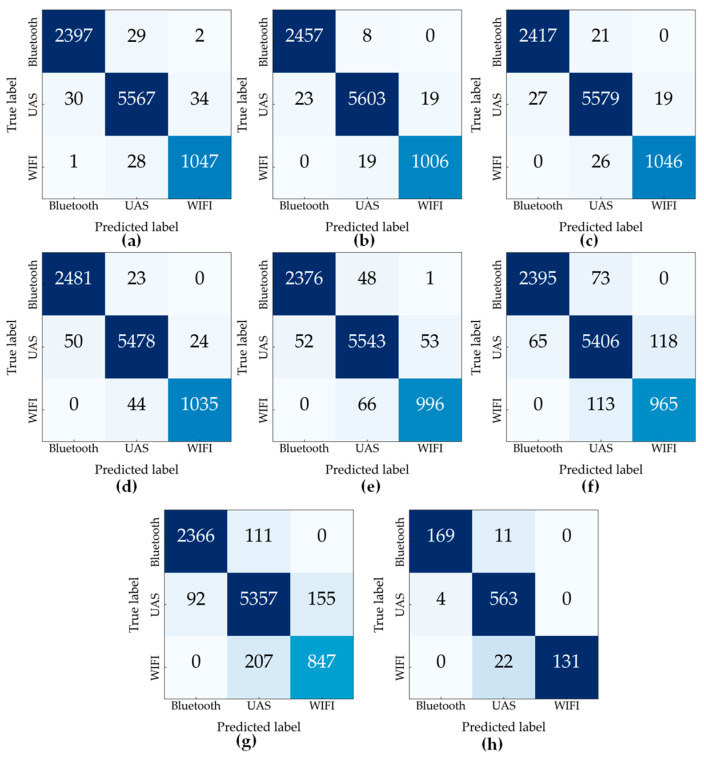
Confusion matrix for detection task at (**a**) 30 dB, (**b**) 25 dB, (**c**) 20 dB, (**d**) 15 dB, (**e**) 10 dB, (**f**) 5 dB, (**g**) 0 dB, and (**h**) unseen SNR.

**Figure 7:**
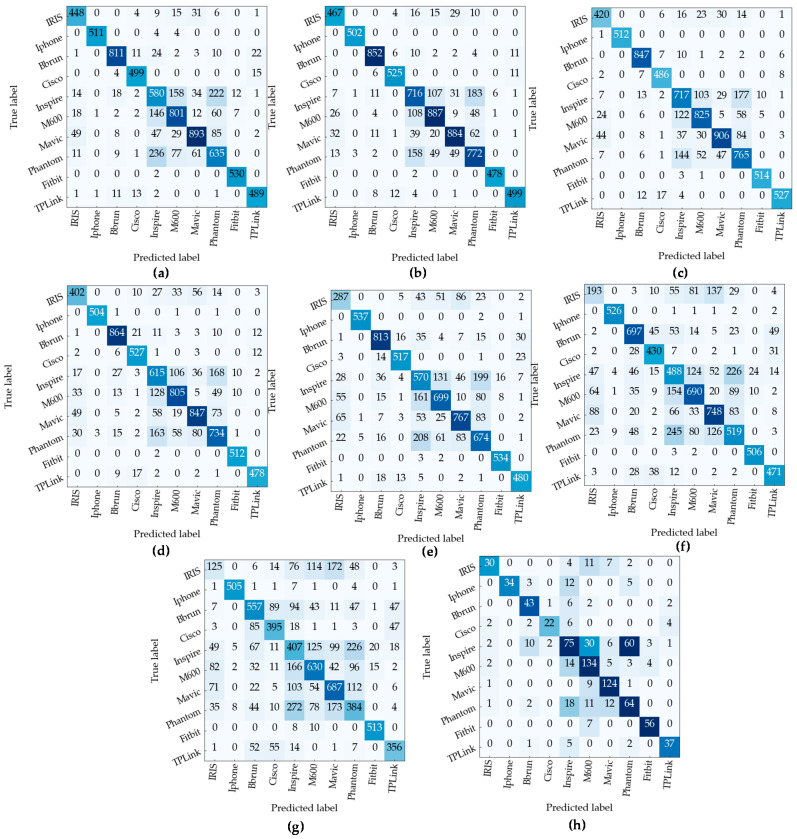
Confusion matrix for device identification task at (**a**) 30 dB, (**b**) 25 dB, (**c**) 20 dB, (**d**) 15 dB, (**e**) 10 dB, (**f**) 5 dB, (**g**) 0 dB, and (**h**) unseen SNR.

Figure 6 and Figure 7 show the confusion matrix of the proposed model for the detection task and specific identification task, respectively. Test accuracy for identification tasks is 98.64%, 98.63%, 98.62%, 98.45%, 97.59%, 95.96%, and 93.81% for 30 dB, 25 dB, 20 dB, 15 dB, 10 dB, 5 dB, and 0 dB, respectively. The model maintains an accuracy of more than 80% for SNR of 20 dB and above, but the accuracy drops with the increase in the noise level because of the presence of more noise. At 10 dB SNR, the accuracy of the model is 76.16%. The performance of the models was evaluated with a set of unseen data from different unknown noise levels. For the detection task, the accuracy was 95.89%. The confusion matrix of the unseen noise for the detection and identification tasks are shown in Figure 6h and Figure 7h. RF signal detection has a higher accuracy as opposed to the specific device identification task, as the model has a higher rate of misclassifying the UAS signals from the devices that are manufactured by the same maker. That can be confirmed from the confusion matrices, as all DJI UAS signals are clustered in an area.

**Figure 8:**
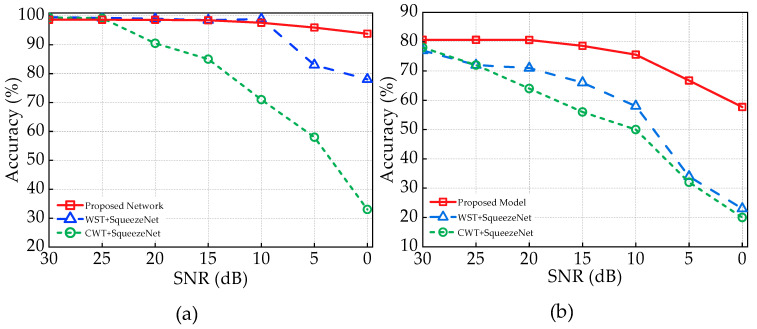
Comparison of models in terms of accuracies with [6] across different noise levels for the (**a**) detection task and (**b**) specific device identification task.

The comparison of the model performance in terms of accuracies with [6] for both tasks is shown in Figure 8. For the detection task, the performance of the proposed model is close to the SqueezeNet architecture exploited in [6] for 30 dB to 10 dB SNR, but with the increase in the noise level, the performance of the SqueezeNet model decreases rapidly, which can be seen in Figure 8a.

After 10 dB SNR, the accuracy of the SqueezeNet model is lower than 90%. However, the proposed model maintains an accuracy of over 93% for all the noise levels discussed in this work. The superior performance of the proposed model can be described because of multiscale architecture. The model extracts features of multiple scales, which assist the proposed model in identifying more prominent features from the noisy data. This shows that the proposed model is more reliable than the SqueezeNet architecture. Figure 8b shows the comparison of the models for the identification task. It can be clearly seen that the proposed model not only outperforms the SqueezeNet but also has a more stable and reliable performance than the methods proposed in ref. [6] for all the noise levels from 0 dB to 30 dB.

Table 6 demonstrates the comparison of average PR, SE, and $F_{1}$ of the proposed model with existing work for RF signals of 30 dB SNR. From the table, it can be said that the proposed model not only outperforms the existing work in terms of accuracy but also in other metrics. For the detection task, the proposed model exhibits a 0.4% and 0.6% higher SE compared to the SqueezeNet with WST and CWT, respectively, which means the proposed model is able to find and correctly classify more of the instances with fewer $FN_{i}$. As $F_{1}$ depends on PR and SE, the model demonstrates a higher $F_{1}$. In the identification task, the model exhibits a 7.55% and 6.25% improvement in precision and a 6.97% and 7.67% enhancement in sensitivity when compared to SqueezeNet with WST and CWT, respectively.

The comparison of accuracies in Figure 8 and other performance metrics in Table 6 demonstrates the superiority of the proposed model in terms of performance.

**Figure 9:**
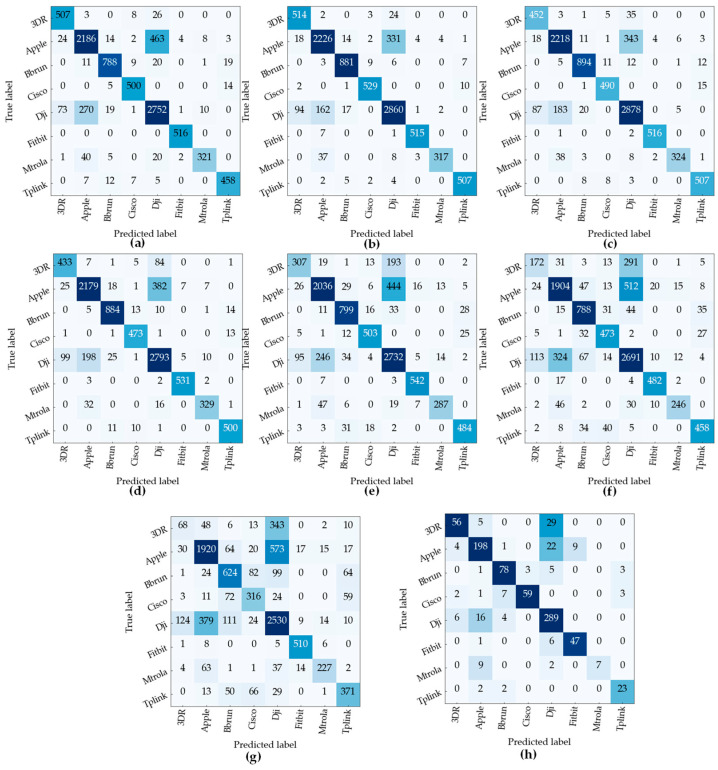
Confusion matrix for device manufacturer identification task at (**a**) 30 dB, (**b**) 25 dB, (**c**) 20 dB, (**d**) 15 dB, (**e**) 10 dB, (**f**) 5 dB, (**g**) 0 dB, and (**h**) unseen SNR.

To address the issue of the higher misclassification among the devices from the same manufacturer observed in Figure 9 the identification model is further modified to classify the devices based on the manufacturers. The four DJI drones and Bluetooth devices from Apple are kept in the same cluster. The performance of the model greatly improves while identifying the signature of the device makers. The overall training and testing of device manufacturer identification are 90.52% and 84.43%, respectively. For the signals from unseen SNR, the accuracy of the model is 84.1%, and for 30 dB to 15 dB, the accuracy of the model is above 85%, and for 0 dB, the accuracy is 71%. The confusion matrix in Figure 9 shows the performance of the model for each class, which shows the model’s ability to classify devices from different manufacturers across various different noise levels. Figure 9h shows that the proposed model can identify most of the devices from the unseen noise levels accurately.

### 3.3. Computational Performance of the Proposed Model

Table 7 shows the inference time and the number of parameters of the proposed system compared with the previous work. SqueezeNet requires 180 milliseconds (ms) with CWT and 151 ms with WST. The higher inference time is due to the utilization of manual feature extraction techniques, which are computationally expensive, but our proposed DL-based method, despite having more parameters, demonstrates an inference time of 0.379 ms for the detection task. For specific device identification task, the inference time of the proposed model is 0.343 ms, which is also significantly lower than [6]. The significant improvement in inference time is because the proposed model does not require any manual feature-extraction technique. The multiscale feature-extraction method utilized in this article is sufficient to extract features from the noisy RF signal.

From the table it is evident that the proposed model offers a reduction in inference time by eliminating the need for feature extraction, which is advantageous for real-time applications.

## 4. Conclusions

In this article, we have utilized an end-to-end deep learning architecture for detecting and identifying UAV signals based on their RF signature. We have considered both UAV and UAV controller signals for our classifier. The communications of the UAV and the flight controller are established at the 2.4 GHz frequency band. Other devices, such as Bluetooth and WIFI signals, also operate in the same range, so we have considered both of these signals as well. Our proposed model is trained on signals from different noise levels, and it can classify signals from unknown SNRs as well, which makes our proposed model more effective. Our proposed model does not require any feature-extraction techniques, which makes it computationally efficient. The raw RF signals, after being normalized, are fed into the network model for training. The model is trained with the data from 0 dB to 30 dB SNR. The average accuracy of the model is 97.53%. Furthermore, the network is evaluated on the data from unseen noise levels to evaluate the performance of the classifier. The overall accuracy for the detection task on unseen data is above 94%. We have obtained an overall accuracy above 76% for specific device identification tasks because of the higher misclassification rate from the same makers. The classification accuracy greatly improves when devices from the same manufacturers are clustered together. The model yields an accuracy of 84% on average when classifying the RF signature of the manufacturers. Finally, we have compared our work with the existing framework and found that the performance of our model, despite having no feature-extraction steps, is more stable across different SNRs.

Our proposed model holds the potential to benefit surveillance systems by effectively detecting and identifying UAS signals in real-time scenarios. The model eliminates the need for manual feature extraction, thus enabling deployment in edge devices. Moreover, its scope of application extends beyond surveillance systems, as it can also be used for image segmentation, feature extraction [25], and video analysis [26] for industries such as health care and others that require similar functionalities. Going forward, we are committed to implementing our model in a diverse range of applications to highlight its versatility and the significant impact it can have across various industries.

## Figures and Tables

**Table 1:** CardRF dataset distribution.

Device Type	Make	Model Name	Number of Signals
UAV and/or UAV controller	Beebeerun	FPV RC drone mini quadcopter	245
	DJI	Inspire	700
		Matrice 600	700
		Mavic Pro 1	700
		Phantom 4	700
	3DR	Iris FS-TH9x	350
WIFI	Cisco	Linksys E3200	350
	TP-link	TL-WR940N	350
Bluetooth	Apple	iPhone 6S	350
		iPhone 7	350
		iPad 3	350
	FitBit	Charge3 smartwatch	350
	Motorolla	E5 Cruise	350

**Table 2:** Configuration table of the proposed model architecture.

Initial Feature Extraction Block			
Layer		Output Volume	
Input		(1024)	
Reshape		(1024, 1)	
Convolution 1D 1		(512, 64)	
ReLU 1		(512, 64)	
MaxPooling		(255, 64)	
Multiscale Feature Extraction Block			
Branch 1		Branch 2	
Layer	Output Volume	Layer	Output Volume
Convolution 1D 2	(255, 64)	Convolution 1D 10	(255, 64)
ReLU 2	(255, 64)	ReLU 10	(255, 64)
Convolution 1D 3	(255, 64)	Convolution 1D 11	(255, 64)
Add 1	(255, 64)	Add 5	(255, 64)
ReLU 3	(255, 64)	ReLU 11	(255, 64)
Convolution 1D 4	(255, 128)	Convolution 1D 12	(255, 128)
ReLU 4	(255, 128)	ReLU 12	(255, 128)
Convolution 1D 5	(255, 128)	Convolution 1D 13	(255, 64)
Dense 1	(255, 64)	Dense 4	(255, 64)
Add 2	(255, 64)	Add 6	(255, 64)
ReLU 5	(255, 64)	ReLU 13	(255, 64)
Convolution 1D 6	(255, 256)	Convolution 1D 14	(255, 256)
ReLU 6	(255, 256)	ReLU 16	(255, 256)
Convolution 1D 7	(255, 256)	Convolution 1D 15	(255, 256)
Dense 2	(255, 64)	Dense 5	(255, 64)
Add 3	(255, 64)	Add 7	(255, 64)
ReLU 7	(255, 64)	ReLU 14	(255, 64)
Convolution 1D 8	(255, 256)	Convolution 1D 16	(255, 256)
ReLU 8	(255, 256)	ReLU 18	(255, 256)
Convolution 1D 9	(255, 256)	Convolution 1D 17	(255, 256)
Dense 3	(255, 64)	Dense 6	(255, 64)
Add 4	(255, 64)	Add 8	(255, 64)
ReLU 9	(255, 64)	ReLU 17	(255, 64)
Averagepooling 1	(127, 64)	Averagepooling 2	(127, 64)
Dropout 1	(127, 64)	Dropout 2	(127, 64)
Terminal Block			
Layer		Output Volume	
Add 9		(127, 64)	
Flatten		(8, 128)	
Dense 7		(3,)/(10,)/(8,)	

**Table 3:** Hyperparameters for model training and evaluation.

Hyperparameters	Values
Train data shape	(51,765, 1024), (51,765, 3) (Detection stage) (43,732, 1024), (43,732, 10) (Specific identification stage) (43,732, 1024), (43,732, 8) (Manufacturer Identification stage)
Test data shape	(9135, 1024), (9135, 3) (Detection stage) (7718, 1024), (7718, 10) (Specific identification stage) (43,732, 1024), (43,732, 8) (Manufacturer identification stage)
Learning rate	0.001
Number of epochs	120
Cost function	Categorical cross-entropy
Activation function	ReLU, softmax
Optimizer	Adam
Batch size	512

**Table 4:** Overall accuracies of the proposed model for different kernel sizes.

Noise Level	Signal Detection Task			Device Identification Task		
	Kernel 3 and 5 (%)	Kernel 3 and 7 (%)	Kernel 5 and 7 (%)	Kernel 3 and 5 (%)	Kernel 3 and 7 (%)	Kernel 5 and 7 (%)
30 dB	98.63	98.64	98.64	80.50	80.51	80.62
25 dB	98.60	98.61	98.63	80.50	80.60	80.61
20 dB	98.20	98.27	98.62	79.49	79.96	80.60
15 dB	98.04	98.36	98.46	78.26	78.39	78.58
10 dB	96.10	96.12	97.59	73.72	74.13	75.58
5 dB	94.65	94.85	96.00	66.29	66.35	66.73
0 dB	92.86	92.88	93.81	55.29	56.50	57.70
Unseen	91.33	91.40	95.88	66.20	67.45	68.78
Overall	97.00	96.60	97.53	74.00	75.54	76.42

**Table 5:** Overall classification performance of the proposed model.

Signal	ACC (%)	PR (%)	SE (%)	$F_{1}\;\left(\%\right)$
Bluetooth	98.95	98.16	98.02	98.5
UAS	97.53	98.06	98.0	98.0
WIFI	98.53	93.23	94.23	93.72

**Table 6:** Comparison of models in terms of various performance metrics.

Method	Detection Task			Identification Task		
	PR (%)	SE (%)	$F_{1}\;\left(\%\right)$	PR (%)	SE (%)	$F_{1}\;\left(\%\right)$
SqueezeNet + CWT [6]	99.70	99.0	-	77.40	76.50	-
SqueezeNet + WST [6]	99.70	99.20	-	76.10	77.20	-
Proposed Model	99.70	99.62	99.60	83.65	84.17	83.88

**Table 7:** Computational and time complexity of the proposed model.

Method	Detection Task (ms)	Identification Task (ms)	Number of Parameters
SqueezeNet + CWT [6]	180	190	722,374
SqueezeNet + WST [6]	151	159	
Proposed Model	0.379	0.343	2,444,928

## Data Availability

All the data used in this study are obtained from public datasets. Readers should be able to obtain these data by requesting the dataset sources described in this study.
